# Proof of concept for developing novel feeds for cattle from wasted food and crop biomass to enhance agri-food system efficiency

**DOI:** 10.1038/s41598-022-17812-w

**Published:** 2022-08-10

**Authors:** Zhengxia Dou, John D. Toth, Dipti W. Pitta, Joseph S. Bender, Meagan L. Hennessy, Bonnie Vecchiarelli, Nagaraju Indugu, Ting Chen, Yunyun Li, Rachel Sherman, Jonathan Deutsch, Bo Hu, Gerald C. Shurson, Brianna Parsons, Linda D. Baker

**Affiliations:** 1 Department of Clinical Studies, School of Veterinary Medicine, University of Pennsylvania, 382 W. Street Rd., Kennett Square, PA 19348 USA; 2 School of Environmental Engineering, Zhejiang Gongshang University, Jianggan District, 18 Xuezheng St, Hangzhou, 314423 China; 3 Institute of Geographic Sciences and Natural Resources Research, Chinese Academy of Sciences, 11A Datun Rd, Chaoyang District, Beijing, 100101 China; 4 Drexel Food Lab, Department of Food and Hospitality Management, Drexel University, 3141 Chestnut St., Philadelphia, PA 19104 USA; 5 Department of Bioproducts and Biosystems Engineering, Colleges of Science and Engineering and Food, Agricultural and Natural Resource Sciences, University of Minnesota, 1390 Eckles Ave., St. Paul, MN 55108 USA; 6 Department of Animal Science, College of Food, Agricultural and Natural Resource Sciences, University of Minnesota, 1420 Eckles Ave., St. Paul, MN 55108 USA

**Keywords:** Environmental sciences, Environmental impact

## Abstract

Modern agri-food systems generate large amounts of crop-based biomass that are unfit for direct human consumption but potentially suitable for livestock feeding in production of meats, milk, and eggs. This study aims to develop novel feeds for cattle from some of those biomass materials through the natural microbial-driven processes of ensiling. Fruit and vegetables resembling supermarket discards were ensiled alone or co-ensiled with corn crop residues, mushroom wastes, etc. via laboratory experiments. Longitudinal sample analyses showed that (co-)ensiling was successful, with pH and fermentation acids changing rapidly into desirable ranges (pH < 4.5, the acids 5–13% DM with lactic acid dominating). The (co-)ensiled products had key nutritional parameters comparable to those of good quality forages commonly used on dairy farms. Additionally, in vitro incubation experiments indicated that the ensiled products could substitute certain conventional feeds while maintaining diet digestibility. Findings from this pilot study provide a proof of principle that quality novel feeds for cattle can be generated by co-ensiling food discards and low-value crop residues. Future research and animal feeding trials to demonstrate the utility of this approach can help societies more effectively utilize untapped biomass resources, strengthening the regenerative capacity of agri-food systems towards a more sustainable food future.

## Introduction

The global food system has and will continue to weather unprecedented challenges in multiple dimensions. At the core are fundamental issues of meeting ever-growing demand in terms of food availability and equity^1–3^ along with the pressing needs to mitigate food’s footprints on climate change, environmental degradation, and unsustainable resource extraction^4,5^. In this context, strategies that promote circularity and expand the regenerative capacity of the agri-food systems are of paramount importance^6,7^. Toward this end, recovery and re-use of biomass materials that are already produced in primary production but ‘lost’ from the linear food supply chain present a unique opportunity. These biomass materials exit the food supply chain mainly as indigestible, unpalatable, or undesirable biomass (IUUB) typically unfit for direct human consumption. Oftentimes, these materials are still rich in carbohydrates, proteins, and other macro- or micro-nutrients^8,9^. These nutrients can be upcycled via livestock feeding, because of farm animals’ innate capability to digest a wide variety of biomass. Therefore, optimizing the use of IUUB materials through livestock presents a viable solution for producing more food with less resource-, environment-, and climate-burdens.

There are three broad categories of IUUB materials^10^: (i) *crop residues* upon removal of human-edible parts, typically inedible/indigestible; (ii) *processing byproducts* (or co-products) consisting of the remaining residues from food/beverage processing industries, generally unpalatable; (iii) *food waste/discards* from various stages of the food supply chain, usually undesirable to humans. Tremendous amounts of IUUB materials are routinely used as feedstuffs, totaling 1140 million metric tons (MT) of crop residues and 600 MT processing byproducts annually worldwide^11^. Still, very large amounts remain untapped resources for livestock-based upcycling. This is particularly true for food waste/discards, which is estimated to be 1300–1600 MT globally^12,13^ and projected to increase in coming decades^14^.

Feeding food waste to animals has had a long history, but the practice has become less common in modern production systems^9,15^. Among the reasons are the economics of precision feeding^16^, the inherent variability in nutritional attributes of food wastes from diverse sources^17^, concerns over disease transmission ^18^, and undervaluation of impacts of food waste on society and sustainability^19^. With the pressing challenges of sustainable food security amid climate change, there have been renewed interests in transforming food waste into feeds for livestock^9,20,21^. It has been demonstrated that proper thermal treatment can render post-consumer food waste (from restaurants and homes) safe for monogastric species; risk of transmission of pathogens and parasites is minimal^18^. Japan and South Korea reportedly convert and utilize 36% and 45% of such food waste for animal feeding^22–24^. Notably, food waste is not created equal^20^. Pre-consumer plant-based food discards would be more suitable for feeding ruminants. For example, feeding studies have demonstrated that marketplace fruit and vegetable discards could replace up to 50% of concentrate feeds in the control diet to support satisfactory steer growth^25^, or substitute 6–18% of concentrate feeds to support milk production (25 kg day^−1^) of Holstein cows^26^. Interestingly, there remains a huge opportunity as wasted fruit and vegetables constitute the single largest food waste stream globally and nationally in the United States^27–29^. To exploit the opportunity requires scientific and management intervention because fresh fruit and vegetable discards are prone to spoilage, which can reduce palatability and nutritional value and increase risks of microbial contamination^30^. Therefore, finding ways to extend the storage life and conserve the nutrients for safe feeding is essential.

Here, we report the efficacy of preserving fruit and vegetable discards via ensiling. Ensiling is a microbial-driven process commonly used on dairy farms for the very purpose of preserving freshly harvested feed crops (around 35% DM) for prolonged storage and feeding. But data are scarce on the utility and robustness of ensiling fruit and vegetable discards, which are much wetter (moisture around 85%, compared to 65% for feed crops typically ensiled). We hypothesized that fresh fruit and vegetables can be co-ensiled with crop residue biomass to produce high quality feeds for cattle. To test the hypothesis, we conducted a series of laboratory experiments in which fresh fruit and vegetables were ensiled alone or in combination (i.e. co-ensiling) with plant biomass such as corn crop residues or spent mushroom compost (see Methods for details). The overarching goal of our research is to develop viable solutions for optimal utilization of IUUB materials, contributing to sustainable livestock production toward enhanced regenerative agri-food systems. Specific objectives included: (i) assess the feasibility of ensiling fresh fruit and vegetables (FFV) alone, or co-ensiling with various biomass substrates via longitudinal studies, (ii) determine key nutritional characteristics of ensiled products, and (iii) evaluate the digestibility of ensiled products via in vitro incubation experiments.

## Results and discussion

### Ensiling efficacy

**Figure 1:**
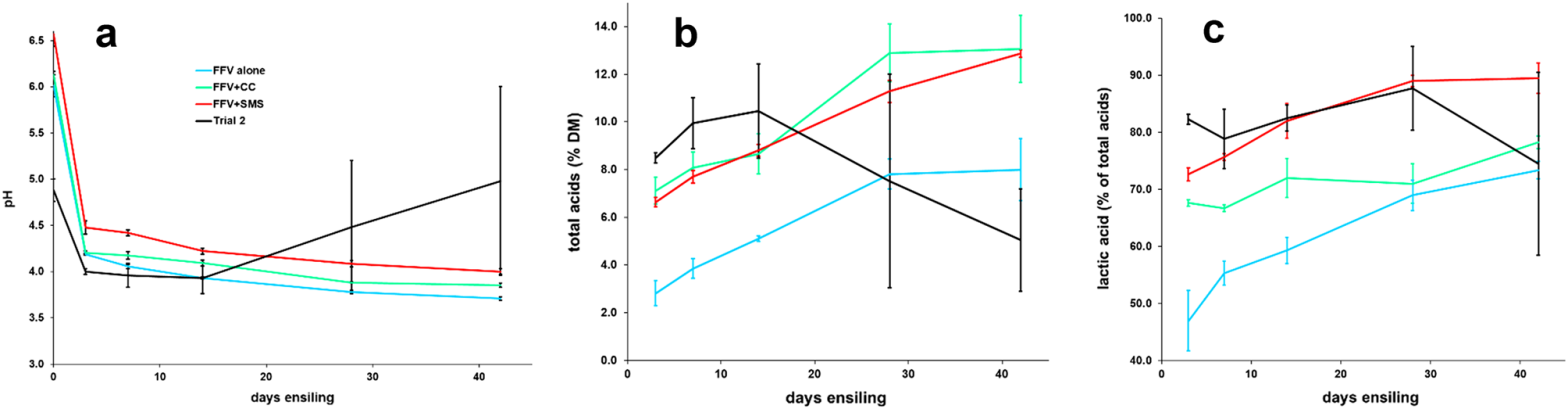
Ensiling parameters pH (**a**), volatile fatty acids (**b**), and lactic acid as a percentage of the volatile fatty acids (**c**) in longitudinal samples collected on days 3, 7, 14, 28, and 42. Abbreviations: FFV, fresh fruits and vegetables; CC, corn cobs; SMC, spent mushroom compost. Error bars are ± 1 standard deviation about the mean. Trial 2 was co-ensiling of FFV with corn stalks, mushroom stumps, spent mushroom compost and wet brewers’ grains.

Three ensiling trials were conducted (see Methods and Supplementary Tables 1 and 2 for details). In Trial 1, ensiling FFV alone (a mixture of 10 types of fruit and vegetables, see Methods) was effective, where pH dropped to 4.2 by day 3 and maintained in a narrow range of 3.7–4.1 thereafter (Fig. 1a). Volatile fatty acids (VFAs) from fermentation increased steadily from 7.1% DM (day 3) to 13.1% (day 28) and maintained near the highest value on day 42 (Fig. 1b). Both pH and VFAs were within the desirable ranges (3.5–4.5 pH, 10–14% DM) for effective preservation of fresh-cut crops such as corn silage^31,32^. Not surprisingly, a considerable amount of liquid accumulated at the bottom of the ensiling vessel; about 110–150 mL per vessel (or 160–210 mL per kg of ensiling substrate) were collected on day 42 (i.e. end of ensiling trial) by gravity drainage. The liquid samples contained 7% DM, with soluble carbohydrates comprising 39.5% of DM. The samples were also enriched in P and Na concentrations but had lower pH, compared to the liquid sample that seeped from the bulk materials of diced FFV during ensiling preparation (i.e. day 0; see Methods). Clearly, proper effluent management is required if FFV is to be ensiled alone on farms.

Co-ensiling FFV with corn cobs (CC) or spent mushroom compost (SMC), as part of Trial 1, had key parameters falling within the desirable ranges as well (Fig. 1). A sharp drop in pH (from 6.0–6.6 to 4.2–4.5) by day 3 was followed by gradual decrease then steady maintenance around 3.9 and 4.0. VFAs increased from 7 to 13% DM (day 3 to day 42) for FFV + SMC, which closely mirrored that of FFV alone (Fig. 1). For FFV + CC, VFAs concentrations were considerably less (3% and 8% DM on day 3 and day 42), but still adequate to attain and maintain pH in a desirable range. It is not clear what might have hindered acids from attaining greater amounts in FFV + CC. Interestingly, FFV + SMC had pH consistently higher (by 0.2–0.3 unit) than that of FFV alone or FFV + CC throughout the ensiling trial, despite greater amounts of the acids (Fig. 1). This is probably due to the SMC substrate having a higher buffering capacity.

In Trial 2, FFV was co-ensiled with corn stalks (CS), mushroom stems (MS), wet brewers’ grains (WBG), and SMC, all originated from local sources (Methods; Supplementary Tables 1 and 2). Ensiling progressed normally in the first two weeks (days 3, 7, 14), with pH and VFAs patterns similar to treatments in Trial 1 (Fig. 1). However, samples obtained on day 28 and day 42 had mean pH higher (4.4 and 5.0) and VFAs lower (8% and 5%) than the earlier-day samples (Fig. 1). We noticed that one of the three replicates on day 28 and day 42 actually had pH and the acids within or close to normal range (3.7 and 4.3 for pH and 12% and 7% for total acids); we thus suspect some mishaps with the possibility of breached anaerobic conditions involving the other ensiling vessels.

Lactic acid, as the most desirable acid among the various fermentation acids generated from the ensiling process, was produced in the greatest quantities throughout the experiment in all cases (Fig. 1c). The ensiled products (i.e. day 42 samples) had lactic acid concentrations averaging 2–5% of DM and 73–90% of total fermentation acids. Acetic acid was 11–26% of VFAs with concentrations of 0.7–2.9% DM, which is within the acceptable range of 1–4% DM for corn silage^31–33^. Propionic acid was present at low concentrations (0.2–0.4% of DM) in some samples, mostly the co-ensiling in Trial 2. Butyric acid or isobutyric acid were not detected in any of the samples.

Trial 3 was conducted to generate ensiled products for use in subsequent in vitro digestibility experiment (Methods; Supplementary Table 1). The ensiled products FFV + CS and FFV + CS + WBG had pH in the desirable range (mean pH 3.9 and 4.0). However, FFV + CS + WBG had acetic acid (instead of lactic acid) dominating (71% of VFAs). We do not know the mechanisms that led to the high level of acetic acid formation. In conventional (corn, legume, grass) silage making, low to moderate amounts of acetic acid can be beneficial because it inhibits yeasts, resulting in improved stability when silage is exposed to air during the feeding phase^34^. However, high concentrations of acetic acid produced during ensiling generally indicates suboptimal fermentation conditions^35^. Nevertheless, when consumed by ruminants, acetic acid in silage can be absorbed from the rumen and utilized for energy by cows^34^.

The fundamental purpose of ensiling is to preserve the biomass that is otherwise susceptible to spoilage, whether it is fresh-cut forage crops or fruit and vegetable discards such as in our case. Listed in Table 1 are established goals and characteristics of good quality corn, legume, and grass silages. Comparatively, the ensiled products in our study had all fermentation parameters (pH, VFAs, and NH_3_-N) falling within the ranges of desirable values, except for FFV + CS + WBG with acetic and propionic acids exceeding the references.

**Table 1:** Fermentation parameters of ensiled products in the present study, as compared to those ‘established goals’ as well as typical values for conventional corn silage, legume silage, or grass silage^a^.

Analyte	Corn silage		Legume / Grass silage			The present study					
	Goal	Typical range	Goal	Typical range		Trial 1			Trial 2	Trial 3	
		(28–32% DM)		Legume silage (28–32% DM)	Grass silage (32–36% DM)	FFV	FFV + CC	FFV + SMC	FFV + CS + SMC + WBG + MS	FFV + CS	FFV + CS + WBG
pH	3.9	3.88	< 4.5	4.91	4.57	3.7 ± 0.02	3.9 ± 0.02	4.0 ± 0.03	4.0 ± 0.5	3.9 ± 0.01	4.4 ± 0.09
Lactic acid (%DM)	4–7%	5.16%	4–7%	4.87%	4.72%	10.2 ± 1.0	5.9 ± 0.9	12.0 ± 0.8	4.0 ± 1.6	5.9 ± 0.4	*1.2* ± *0.6*
Acetic acid (%DM)	< 2%	3.49%	< 3%	3.80%	2.05%	2.9 ± 0.4	2.1 ± 0.4	1.4 ± 0.4	0.7 ± 0.4	4.2 ± 0.3	*6.2* ± *0.8*
Propionic acid (%DM)	< 0.5%	0.35%	< 0.5%	0.33%	0.13%	n.d.^b^	n.d	n.d	0.2 ± 0.1	n.d	*0.9* ± *0.07*
Butyric acid(%DM)	< 0.01%	0.03%	< 0.1%	0.91%	0.34%	n.d	n.d	n.d	n.d	n.d	n.d
Total acids(%DM)		9.05%		9.9%	7.2%	13.1 ± 1.4	8.0 ± 1.3	12.9 ± 0.2	*5.1* ± *2.1*	10.1 ± 0.6	8.4 ± 0.8
Lactic acid (% total acids)	65–70%	57.25%	65–70%	49.1%	65.2%	78.2 ± 1.1	73.4 ± 1.6	89.5 ± 2.7	74.5 ± 16.0	*58.4* ± *0.8*	*16.5* ± *7.0*
NH_3_-N (% total N)	< 7%	8.58%	< 10%	16.4%	9.12%	2.2 ± 0.4	2.5 ± 0.7	1.1 ± 0.1	1.4 ± 0.8	1.7 ± 0.04	1.6 ± 0.04
1,2 Propanediol (when present)		1.30%				n.d	n.d	n.d	n.d	n.d	n.d

^a^Data for the goals and conventional silages were from Ward and de Ondarza^35^.

^b^Abbreviations: FFV for fresh fruit and vegetables; CC for corn cobs; CS for corn stalks; SMC for spent mushroom compost; WBG for wet brewers’ grains; MS for mushroom stems, and n.d. for analyte not detected. Deviations from reference values are in italics.

Conventional silage-making requires substrate dry matter to be > 30–32% to minimize the risks of *Clostridia* growth. *Clostridia* bacteria are one of the most common undesirable bacteria that may persist in unstable silage, leading to higher dry matter loss and poor silage palatability^34,35^. In our study, the wet substrates (< 25% DM in all co-ensiling treatments; Supplementary Table 1) did not succumb to *Clostridia* growth because no butyric acid was detected (*Clostridia* bacteria convert lactic acid to butyric acid). The rapid decrease in pH within the first few days of fermentation might have inhibited *Clostridia* growth, which requires pH 4.5 or above^36^. It is also possible that the substrates (FFV and the other biomass) were not laden with *Clostridia* bacteria, unlike forage crops harvested directly from agricultural fields that may be contaminated with soil.

### Nutritional attributes of ensiled products

Crude protein (CP) concentrations in ensiled products ranged from 6.9% to 18.1% DM (Table 2). Compared to FFV alone, the addition of corn crop residues diluted CP content whereas wet brewers’ grains and mushroom stumps elevated CP in the co-ensiled products. Soluble protein concentration was highest in ensiled FFV alone (63.1% CP) but considerably less in most of the co-ensiled products (27.2–50.4% CP). Protein bound to ADF and NDF ranged from 2.7 to 22.5% (Supplementary Table 3). The ADF-bound protein is indigestible in ruminants and passes into manure. The difference between NDF- and ADF-bound protein provides a measure of bypass protein for intestinal degradation and absorption. Compared with conventional forages typically used in feeding dairy cows, the ensiled products of FFV alone or in combination with corn residues (CC, CS) had CP levels similar to that of corn silage (around 8.5% DM), whereas co-ensiling FFV with WBG, MS, and SMC led to ensiled products with CP levels close to that of grass or alfalfa hay (13–20% DM, Table 2).

**Table 2:** Summary of key nutritional parameters of ensiled products, as compared to those of corn silage, legume silage, and grass silage.

Parameter	FFV^a^	FFV + CC^a^	FFV + SMS^a^	Trial 2 mixture	FFV + CS^a^	FFV + CS + WBG^a^	Corn silage^b^	Alfalfa hay^b^	Grass hay^b^
CP^c^ (% DM)	10.5	7.4	13.8	18.1	7.6	16.0	8.0	19.7	13.6
SP^d^ (% CP)	65.3	50.4	30.2	27.2	41.2	32.6	61.7	40.1	29.5
ADF (% DM)	13.6	36.5	20.4	39.2	40.4	37.7	26.1	32.8	34.9
NDF (% DM)	15.9	57.2	32.4	48.0	57.8	55.4	39.7	41.6	54.4
Lignin (% DM)	2.3	4.8	18.7	10.1	6.4	6.4	2.8	7.6	5.2
Sugar (% DM)	38.6	− ^e^	− ^e^	1.3	1.0	0.5	1.1	9.7	8.2
Starch (% DM)	− ^e^	− ^e^	− ^e^	5.1	6.9	8.4	35.5	1.6	2.6
TDN	− ^e^	− ^e^	− ^e^	53.0	54.9	59.0	70	52	52
Crude fat (% DM)	8.8	− ^e^	− ^e^	− ^e^	1.6	4.2	3.2	2.9	3.1
Ash (% DM)	8.0	3.8	21.5	3.9	8.3	8.0	4.3	10.0	6.0

^a^Abbreviations: same as Table 1 footnotes. ^b^Values for the conventional dairy feed silages are from the feed dictionary of UPenn Dairy Ration Analyzer. ^c^Crude protein. ^d^Soluble protein as fraction of crude protein. ^e^Data not available.

Fibrous feedstuffs are indispensable in ruminant diets for maintaining normal rumen fermentation and rumination, lowering the risk for rumen acidosis and post-calving disorders ^37,38^. In our study, ensiled FFV alone had lower ADF (13.6%) and NDF (15.9% DM) concentrations relative to conventional forages. Co-ensiled products had ADF concentrations in the range of 20.4–40.4% DM and NDF concentrations 32.4–57.8%, which are comparable to those found in grass or alfalfa hay (Table 2). The ensiled FFV + SMC had high lignin content (18.7% DM), originating from the raw SMC substrate (32.6% lignin; Supplementary Table 2). Higher lignin content of forages is generally associated with lower amounts of nutrients for the animal. Restricting FFV + SMC to a relatively low ratio in a total mixed ration/diet can help limit the dietary lignin level to within an acceptable range. Future research is needed to evaluate the use of mechanical processing to differentiate various fiber fractions in raw SMC materials. Additionally, the high ash content in FFV + SMC (21.5% DM) could also be lowered via dilution in total mixed rations and/or SMC substrate processing. Ash content in other ensiled products evaluated in this study were mostly < 10% DM (Table 2). Conventional silages generally have ash concentrations of about 4.4% (corn silage) and 10.9% (legume silage;^35^).

The parameter of total digestible nutrients (TDN) is the sum of digestible fiber, protein, lipid, and carbohydrate components of a feedstuff. It is the simplest form of energy evaluation of a feed, directly related to digestible energy of the feed^39^. High quality forages such as alfalfa have TDN typically ranging from 50 to 60% DM while low quality forages range from 40 to 50% ^40^. Accordingly, ensiled products evaluated in our study had TDN concentrations ranging from 50 to 60% which are equivalent to TDN content commonly observed in medium–high quality forages (Table 2).

### Feed hygiene of ensiled products

Ensiled products tended to be ‘cleaner’ (lower molds and yeast counts) than day 0 samples (Table 3). Mold counts were within the range considered safe (< 5.7 log_10_ CFU g^−1^) or relatively safe (< 6.0 log_10_ CFU g^−1^) for conventional feeds (https://www.foragelab.com/Services/Forage-and-Feed/Mold-and-Yeast-Evaluation/), except for FFV + CC. In the latter case, a crack in the lid for one of the three ensiling vessels was observed at the end of the experiment, indicating a probable breach of anaerobic conditions and thus resulting in greater mold growth (mean value 6.48 log_10_ CFU g^−1^). Higher than acceptable mold counts may lead to depressed digestibility, feed intake, and animal performance. *Fusarium* and *Mucor* species were the predominant mold species identified in the samples. *Fusarium* is a large genus of filamentous fungi widely distributed in soil and plants; some have been reported to produce mycotoxins^41^. *Mucor* does not produce mycotoxins^42^. Yeast concentrations in our samples were considered acceptable for well-preserved conventional silage (< 4 log_10_ yeast CFU g^−1^;^43^), except for the ensiled product of Trial 2 (Table 3) presumably due to experimental mishap as mentioned earlier.

**Table 3:** Mean and standard deviation of mold and yeast analyses on selected samples from ensiling experiments^a^, values are log_10_ CFU g substrate^a^.

	Mold counts				Yeast counts			
	FFV	FFV + CC	FFV + SMC	Trial 2	FFV	FFV + CC	FFV + SMC	Trial 2
Day 0	2.82 ± 2.46	4.47 ± 4.27	4.22 ± 4.11	–	3.22 ± 3.31	6.91 ± 6.68	6.10 ± 5.82	–
Day 42	2.70 ± 0.0	6.48 ± 6.64^b^	3.78 ± 3.85	5.76 ± 0.42	2.82 ± 2.46	3.00 ± 2.94	2.70 ± 0.0	7.13 ± 0.99
**Mold counts concerning animal feeding**^**c**^								
Mold count (log_10_)		Guidance						
< 5.7		Safe						
5.7–6.0		Relatively safe						
6.0–6.3		Discount energy (0.95), feed with caution						
6.3–6.5		Discount energy (0.95), closely observe animals and performance						
6.5–6.7		Discount energy (0.95), closely observe animals and performance, dilute with other feeds						
> 6.7		Discontinue feeding						

^a^Abbreviations: same as Table 1 footnotes. ^b^High mold count in one ensiling vessel due to crack in lid and air entry. ^c^Source: CVAS (https://www.foragelab.com/).

### In vitro* incubation outcomes*

In vitro incubation is a commonly employed method that uses rumen fluid to digest feed samples in incubation vials; fermentation parameters are determined to assess the digestibility of treatment diets when exposed to rumen microbes, ultimately helping to predict potential impact on animal performance. After 24 h incubation in our study, all treatments had fermentation parameters characterized by decreases in pH (by 1.8–1.9 unit), increases in ammonia-N concentration (by up to 2.8 mg dL^−1^), and changes in VFAs makeup, compared to 0 h (Supplementary Tables 4 and 5). Additionally, gas production was in the range of 86–106 mL per vial. Such gas is generally a mixture of methane and carbon dioxide plus trace amounts of other compounds. Future investigation to determine the amount and ratio of methane in the gaseous emissions can provide further insights regarding carbon footprint mitigation related to novel feeds in diet. Taken together, our results suggested normal fermentation activities taking place in the incubation vials, as treatment diets did not differ from the control diet in terms of gas production, ammonia-N concentration, and VFAs in most cases, with pH slightly higher (by < 0.07 unit on average; Table 4). In essence, there were little differences in digestibility between the control and treatment diets containing novel feeds. This implies that the novel feeds could potentially substitute conventional feeds (5% or 10%) to support animal requirement, although actual cow performance remains to be determined experimentally via feeding studies.

**Table 4:** In vitro fermentation parameters after 24 h incubation. Values are means of three replicates ± one standard deviation; the same letters following a parameter value in a row are not significantly different using Fisher’s protected least significant difference test (Pr > F) at a probability level of 0.05.

Analyte	Unit	Diet^a^						
		TMR	TMR + 5%NF1	TMR + 10%NF1	TMR + 5%NF2	TMR + 10%NF2	TMR + 10%NF1 + C + P	TMR + 10%NF2 + C + P
pH		5.16 ± 0.01c	5.21 ± 0.02b	5.25 ± 0.02a	5.23 ± 0.01ab	5.25 ± 0.04a	5.20 ± 0.02b	5.22 ± 0.02ab
Gas production	mL	106 ± 3a	103 ± 4a	104 ± 5a	99 ± 5a	86 ± 23a	77 ± 29a	88 ± 25a
NH_3_-N	mg dL^-1^	11.65 ± 0.74a	10.70 ± 0.65ab	9.95 ± 0.22bc	11.95 ± 0.84a	11.33 ± 1.91ab	8.49 ± 0.33c	11.91 ± 0.98a
Acetic acid	% mmol	48.37 ± 1.25a	50.21 ± 2.14a	50.22 ± 1.04a	50.29 ± 1.83a	50.78 ± 1.01a	48.52 ± 1.71a	50.37 ± 1.79a
Propionic acid	% mmol	30.25 ± 0.43ab	29.45 ± 0.75bc	29.26 ± 0.50c	29.19 ± 0.61c	28.98 ± 0.46c	30.44 ± 0.49a	29.50 ± 0.52abc
Butyric acid	% mmol	16.49 ± 0.73a	15.52 ± 0.85a	15.52 ± 0.52a	15.62 ± 0.84a	15.12 ± 0.59a	16.31 ± 0.85a	15.42 ± 0.91a
Isobytyric acid	% mmol	0.74 ± 0.08a	0.73 ± 0.18a	0.73 ± 0.08a	0.69 ± 0.08a	0.82 ± 0.14a	0.87 ± 0.09a	0.73 ± 0.15a
Isovaleric acid	% mmol	1.78 ± 0.06a	1.80 ± 0.20a	1.91 ± 0.07a	1.87 ± 0.20a	1.95 ± 0.03a	1.79 ± 0.21a	1.77 ± 0.16a
Valeric acid	% mmol	2.36 ± 0.12a	2.29 ± 0.22a	2.37 ± 0.11a	2.33 ± 0.19a	2.35 ± 0.08a	2.27 ± 0.17a	2.21 ± 0.16a

^a^Abbreviation: TMR, total mixed ration; NF1, novel feed 1 (ensiled product of fresh fruit and vegetables with corn stalks); NF2, novel feed 2 (ensiled product of fresh fruit and vegetables with corn stalks and wet brewers’ grains); C + P, ground corn plus protein mix.

**Figure 2:**
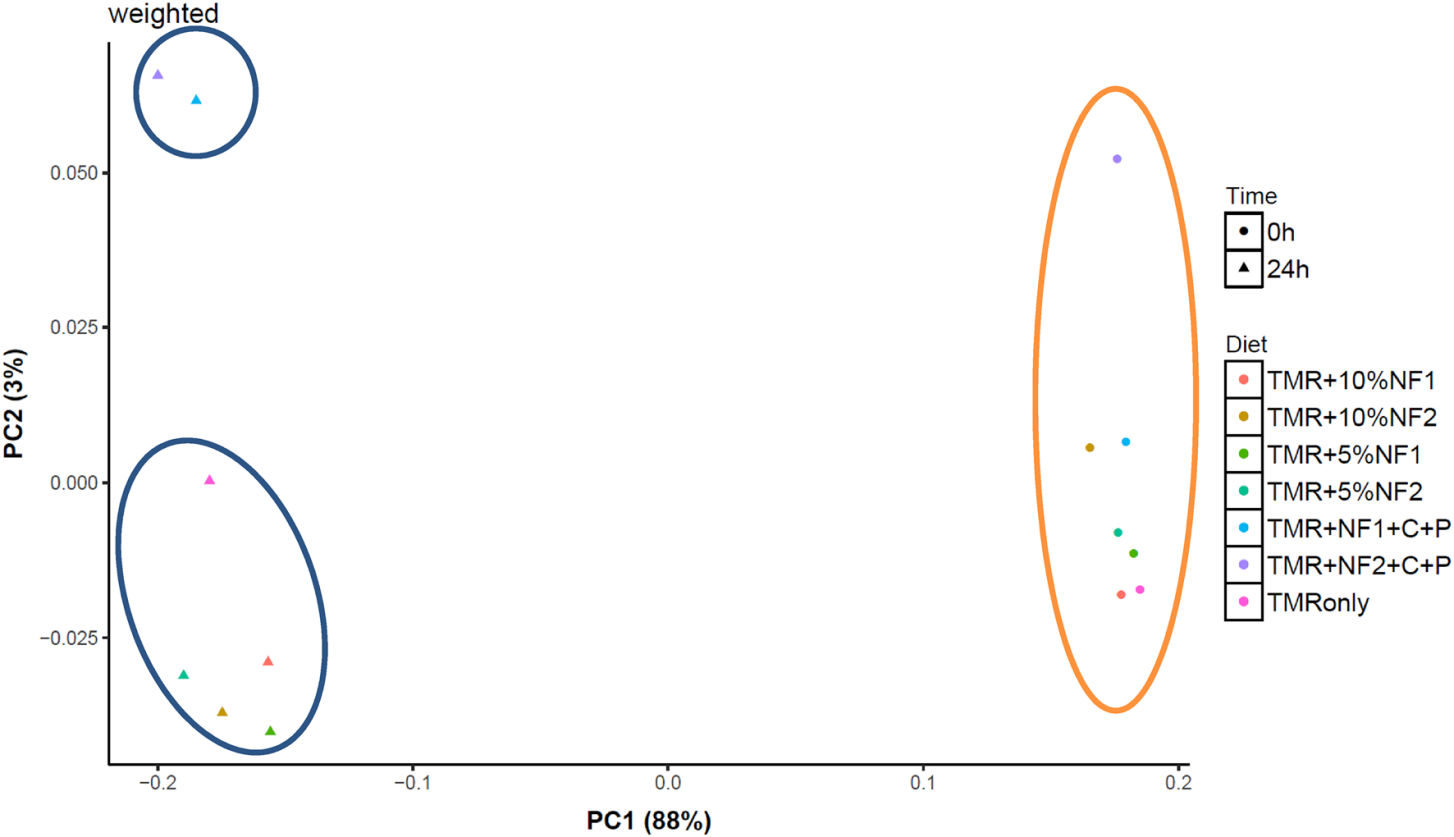
Comparison of bacterial communities in 0 vs. 24 h in vitro incubation samples. TMR for total mixed ration, TMR only served as control; NF1 for ensiled novel feed FFV + CS; NF2 for ensiled novel feed FFV + CS + WBG; C + P for ground corn and protein mixes. See Table 1 footnotes for additional abbreviations.

**Figure 3:**
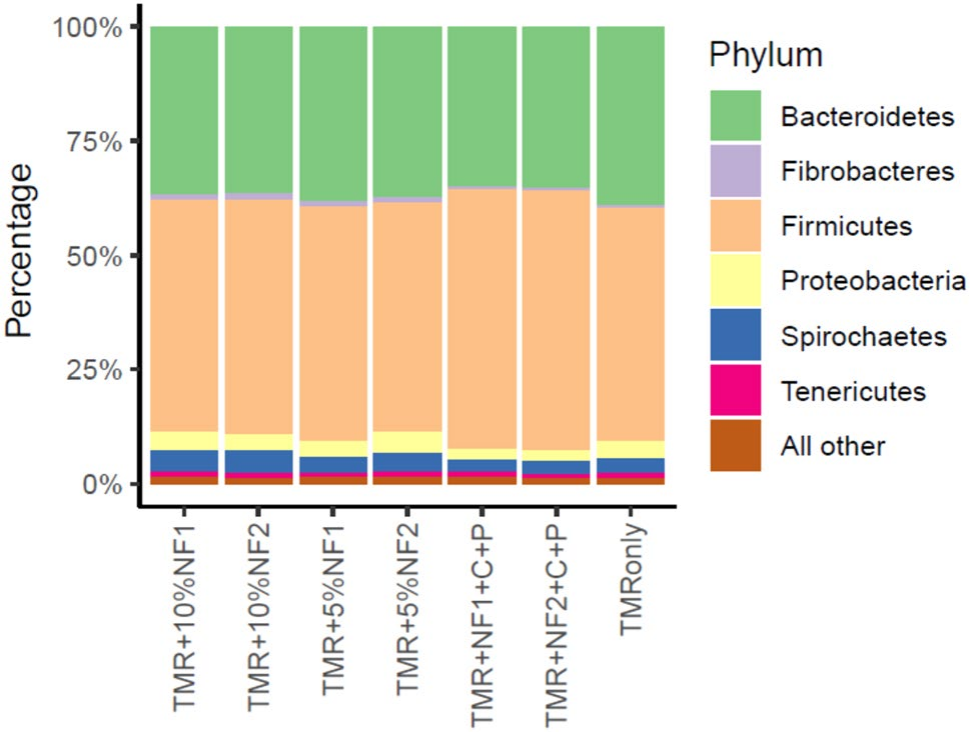
Comparison of individual bacterial phyla after 24 h in vitro incubation. Treatments and abbreviations are the same as in Fig. 2 caption.

Microbial compositions at the community level at 24 h differed dramatically from that of 0 h (Fig. 2), a result of rumen microbes responding to the diets during in vitro incubation. Inclusion of novel feeds alone (5% or 10%) did not change microbial community makeup at 24 h as compared to the control diet, but the addition of C + P apparently triggered some microbial differences as shown in Fig. 2 (PC2 on Y axis). The addition of C + P was to balance dietary nutrients against cow requirements in the ration formulation model (Methods, Supplementary Table 4). At the phylum level, Firmicutes and Bacteroidetes were the most abundant phyla in all treatments, together accounting for 90% of bacterial abundance, whereas Proteobacteria, Spirochaetes, and Tenericutes were less abundant (Fig. 3). The community-level difference between C + P and the other treatment diets were reflected at the phylum level, with the dominating Firmicutes even more abundant (by a few percentage) while Proteobacteria, Spirochaetes, and Tenericutes further less (Fig. 3). For the dominating Firmicutes at the genus level, *Butyrivibrio*, unclassified *Clostridiales*, and *Clostridium* were in higher abundance with the C + P treatments compared to all other treatments (Supplementary Table 6). Taken together, our findings from the in vitro incubation study suggest that the novel feeds at 5% or 10% inclusion rates could maintain diet digestibility, thus supporting milk production. The more nuanced changes in microbial profiles with the addition of ground corn and protein mixes and its potential implications regarding animal productivity as well as carbon flow pathways deserve further investigation. Future studies through actual feeding trials are warranted.

### Perspectives

Previous studies have tested the ensiling of various fruit or vegetable residues, such as cabbage and lettuce leaves, carrot residues, yacon tuber, pineapple residues, or mixed vegetables discarded from marketplaces^44–49^. These studies focused on characterization of ensiled products at the end point. Results from our study with samples collected longitudinally over time allowed better understanding of the ensiling processes of FFV and crop biomass as they progressed, in addition to nutritional attributes of the end products. We found that desirable conditions for preserving the substrates were attained rapidly. More importantly and for the first time to our knowledge, we show that co-ensiling FFV with drier crop residue materials has the synergistic benefits of minimizing silage effluent and retaining soluble nutrients meanwhile making it possible to create value-added quality feeds from crop residues that are otherwise underutilized or under-valued.

Future investigations need to test the utility of making quality novel feeds via scale-up studies to pave the way for eventual on-farm adoption. Animal feeding trials will be essential to document animal responses to diets containing novel feeds regarding key performance parameters such as dry matter intake, milk yield and milk components (protein and fat fractions), as well as health indices. Studies with in-depth evaluation of rumen microbial responses can help shed light on whether novel feeds would modify methanogen profiles and potential implications regarding enteric methane emissions. Further, IUUB materials generated in agri-food systems are diverse and versatile. Studies expanding the scope to test various crop biomass as co-ensiling substrates with fresh produce materials could bring about new upcycling opportunities. For fresh produce discards, particularly unsellable fruit and vegetables from retail markets, understanding the variability (amounts, composition, seasonal flow, etc.) and how it may affect animal performance is very important. Additionally, logistics such as source distribution and transport distance will need to be considered for lifecycle-based assessment of carbon footprints. Comprehensive analyses to address multi-sustainability objectives, e.g. socioeconomic impact, climate change, land-, water-, and nutrient-footprints from novel feeds substituting conventional feed ingredients will be critical to demonstrate broader impacts and to inform resource- and climate-smart policymaking.

At the farm level, feed security with adequate and uninterrupted supply of quality feeds is central to sustain a given livestock operation. A farm’s feed security, or feed insecurity risks, are subject to various factors. Some risks are external and beyond the farmer’s control. Examples include feed supply and price volatility, competition from non-agriculture sectors, or increasing adverse weather events aggravated by climate change leading to crop failure or harvest loss. Developing low-impact non-competing novel feeds from wasted food as well as inexpensive, reliable, locally available crop biomass can help alleviate some of the uncertainties and mitigate relevant risks. This would help enhance farming resilience and benefit societies with nutrient-rich food produced more sustainably.

Global demands for meats, milk, and eggs are expanding, stemming increasingly from developing economies. The livestock sector must strive to meet the growing demands meanwhile addressing sustainability challenges and lowering unintended consequences. Innovative strategies and practices that mitigate feed vs. food competition and leverage livestock to upcycle human-unfit biomass are essential. Findings in our pilot study can help advance the endeavor in developing viable solutions to support sustainable livestock production while strengthening the regenerative capacity of the agri-food system toward a more livable future.

## Materials and methods

### Description of ensiling substrates

Three ensiling trials were conducted for a period spanning over a year. For consistency as well as cross comparison, we created a formula to make fresh fruit and vegetable mixtures with items obtained from a discount produce market to be used in each trial. The formula included ten types of fruit and vegetables that topped the list of unsellable fresh produce in US supermarkets, as reported by Buzby et al.^27,28^. Together, they accounted for 55.3% of the total (by weight) of the national supermarket fruit and vegetable waste data^27^. Our formula consisted of: (i) watermelon, romaine lettuce, apple, potato, and tomato, each making up 14% by weight in the mix, plus (ii) orange, cantaloupe melon, onion, bell pepper, and banana, each 6%.

Substrates for co-ensiling trials included corn cobs (CC), corn stalks (CS), mushroom stumps (MS), spent mushroom compost (SMC), and wet brewers’ grains (WBG), in addition to FFV. The raw materials for CS were obtained from one southeast Pennsylvania dairy farm and CC from another. Corn cobs were sieved to pass 7 mm. Corn stalks were processed first through a silage chopper then ground in a cutting mill to pass 1 mm. Mushroom stumps are the lower part of the stem that is removed and discarded upon harvest and prior to processing and packaging. Spent mushroom compost is the growth media cleared out of the mushroom house after the growing cycles, which consists of remains of the original components such as wheat straw, corn stalks, peat moss, etc.^50^. Both MS and SMC substrates were obtained from a local large-scale mushroom facility. For ensiling preparation, the MS specimen was brush-cleaned of clinging compost materials and the SMC specimen was air-dried and clumps broken down by hand to smaller pieces. The WBG originated from a local brewery and was kept under refrigeration until beginning the ensiling experiment. Analyses of physical, chemical, and nutritional parameters of the raw substrates are in Supplementary Table 2.

### Ensiling trials

Three ensiling trials were conducted. Trial 1 tested the ensiling of FFV alone, co-ensiling of FFV with CC, and co-ensiling of FFV with SMC. Trial 2 tested co-ensiling of FFV with CS, MS, SMC, and WBG. Trial 3 was conducted with the co-ensiling of FFV + CS and FFV + CS + WBG, respectively; the ensiled products were used in a subsequent in vitro incubation experiment. Ratios of substrates in the co-ensiling treatments are listed in Supplementary Table 1; samples obtained at the beginning of the experiments (day 0) had 24–25% DM for all co-ensiling treatments. FFV alone had 12.3% DM.

For each ensiling trial, a fresh batch of FFV was made according to the formula described earlier. Raw items were cut into 14 mm cubes using a commercial food processor (Robot Coupe model CL 50, Robot Coupe USA, Inc., Ridgeland, MS). To avoid clogging the processing unit, hard stems from peppers and bananas as well as rinds from melons were manually cut to approximately 14 mm pieces then added to the mixture. Processed FFV was bulked in a plastic tub, the content was thoroughly mixed by hand upon ensiling preparation.

Ensiling was conducted using 0.95 L polyethylene containers with snap-on lids. The containers and lids were wiped with 70% ethanol immediately prior to filling. Each co-ensiling treatment was prepared by weighing out the substrates into a tub and homogenizing manually. FFV alone or with co-ensiling materials was packed into the ensiling containers, tamped down to eliminate air pockets and filled to the top to limit air-filled head space. Lids were snapped on, with circumferences coated with waterproof silicone sealant to prevent air exchange. To permit gas release while maintaining anaerobic conditions in the vessel, a water-filled fermentation lock was inserted through a rubber grommet on the lid and sealed with silicone sealant. Vessels were placed on a laboratory bench under ambient light and temperature (approximately 20 °C). Preliminary trials indicated that the temperature inside the vessels fluctuated in a narrow range of 18–20 °C during ensiling; temperature was not monitored in subsequent trials.

The longitudinal experiments were conducted for 42 days in Trials 1 and 2; sample collection took place on days 0, 3, 7, 14, 28 and 42. Trial 3 was conducted for 28 days, and samples were collected on day 28 to be used for the in vitro experiment. At each sampling time, three replicates of the vessels per treatment were removed and the ensiling process terminated; the content was emptied into a plastic tub and mixed thoroughly with a sterile plastic scoop for sampling and analysis.

### In vitro* experiment*

In vitro incubation was conducted to evaluate digestibility when the ensiled products were added (as novel feeds) to total mixed ration (TMR) made for lactating cows at the Marshak Dairy. The latter is a 180-cow research and teaching facility at the University of Pennsylvania, School of Veterinary Medicine. The TMR consisted of grass hay, corn silage, triticale, ground corn, proteins, byproducts, minerals and vitamins. Each of the ensiled products (FFV + CS, labeled as novel feed 1, NF1 in short; FFV + CS + WBG, novel feed 2, NF2 in short) was a composite made from equal aliquots of three replicates. All feed samples were oven-dried and ground to pass 2 mm in a high-speed spice grinder. The in vitro incubation experiment consisted of seven treatments, in triplicate, with inclusion rates of 5% and 10% per novel feed, plus ground corn and protein mixes (C + P) added to diets of 10% novel feeds for the purpose of balancing nutrients against cow requirements (Supplementary Table 4).

Rumen fluid was obtained via stomach tubing^51^ from three cows at the Marshak Dairy following IACUC protocols approved by the Office of Animal Welfare at the University of Pennsylvania. Rumen fluid was checked for pH, poured into purged 250 mL bottles, and kept in a warm container until being transferred to the laboratory. At the laboratory, rumen fluid from all three cows was poured into a purged 1 L bottle which was maintained in a water bath at 37 °C under constant flow of CO_2_, to make a pooled inoculum. The inoculum was added to 21 glass vials (seven treatments in triplicate), each containing 0.75 g feed sample and 12 mL of MacDougall’s buffer. To add inoculum, each vial was purged with CO_2_ for 30 s, then 6 mL inoculum was pipetted in, and the vial was purged again for 30 s. Vials were sealed with rubber septa and metal lids and crimped. Once all 21 vials had been filled, 60 mL syringes were inserted into the top for collecting and recording gas production, and the vials were placed into the water bath with gentle agitation at 37 °C for 24 h. Upon completing the incubation, all vials were removed from the water bath, gas volumes were recorded, subsamples (~ 2–3 mL each) were taken to check pH, and the remaining contents in the vial were strained through 4 layers of cheesecloth to separate the solid and liquid fractions. Approximately 500 mg of the solid fraction and 0.75 mL of the liquid fraction from each vial were placed into 2-mL Eppendorf tubes (in duplicate) and stored at − 80 °C until extraction for DNA. Additionally, to prepare samples for VFAs/ammonia analysis, 5 mL of the liquid fraction was spun at 10,000 × *g* for 10 min. Four mL of the supernatant was transferred to a new tube and 800 µL of 36% metaphosphoric acid was added, and the tube was spun at 15,000 × *g* for 20 min. The remaining supernatant was removed and stored in a -20 °C freezer until sending to a certified service laboratory (Cumberland Valley Analytical Services, Waynesboro, PA) for analysis.

The same steps were repeated for 21 control vials with 0 h incubation. After inoculum was added, vials were gently agitated and then immediately processed for sampling following the same procedure described above.

### Sample analysis

For samples collected during the ensiling experiment, a subsample of approximately 75 g was used for gravimetric DM determination using a forced-draft oven (80 ˚C 24 h). Another subsample, 50 g, was used for pH determination (1:1 ratio in deionized water). A third subsample, roughly 400 g, was sent to the same certified laboratory (above) for analyses. The remaining materials were archived in a − 20 °C freezer.

Analyses of ensiling process parameters included concentrations of lactic, acetic, propionic, butyric, and iso-butyric acids plus 1, 2 propanediol, in addition to pH and DM. These analyses were conducted for longitudinal samples collected during the course of the ensiling experiments. Additionally, selected samples were analyzed for a suite of nutritional indices (the “CPM Plus” analytical package by wet chemistry, https://www.foragelab.com/Services/Forage-and-Feed/Chemistry). The nutritional indices included all macro- and micro-nutrients as well as fiber profiles. The selected samples included those ensiled products i.e. at the end of ensiling trial (day 42 or day 28), and in some cases samples obtained at the beginning of experiments (day 0 or day 3). Furthermore, selected samples were analyzed for yeast and mold counts with mold identification. Additionally, liquid effluent from the ensiling of FFV alone was obtained by gravity drainage and analyzed for dry matter, water-soluble carbohydrates and minerals.

For samples obtained from the in vitro incubation, a portion of the liquid fraction from each vial was analyzed at the certified service laboratory (above) for VFAs (acetic, propionic, butyric, isobutyric, isovaleric, and valeric) in addition to ammonia. Genomic DNA was extracted from 250 µL of the liquid fraction and 250 mg of the solid fraction of each incubation vial using the repeated bead beating and column (RBB + C) method followed by extraction with a commercial kit (QIAmp Fast DNA Stool Mini Kit; Qiagen Sciences, Germantown, MD) as described in Yu and Morrison^52^. Extracted DNA was pooled by fraction and treatment and the V1-V2 region of the bacterial 16S rRNA gene was PCR-amplified in triplicate using the bacterial-specific primers F27 (5′-AGAGTTTGATCCTGGCTCAG-3′) and R338 (5′-TGCTGCCTCCCGTAGGAGT-3′) barcoded with a unique 12-base error-correcting Golay code for multiplexing as described in Song et al.^53^. Polymerase chain reaction was performed using the Accuprime Taq DNA Polymerase System (Invitrogen; Carlsbad, CA). Thermal cycling conditions involved an initial denaturing step at 95 °C for 5 min followed by 20 cycles (denaturing at 95 °C for 30 s, annealing at 56 °C for 30 s, extension at 72 °C for 90 s) and a final extension step at 72 °C for 8 min. Amplicons from each sample were combined and each library was added to a pool in equimolar concentration. The final pool was bead-purified using Agencourt AMPure XP Beads (Beckman Coulter, Brea, CA). Sequencing was performed at the PennCHOP Microbiome Core using the Illumina MiSeq platform.

### Bioinformatics and data analysis

Ensiled sample results for nutritional, ensiling process, and mold/yeast evaluation parameters reported by the certified laboratories were entered in a Microsoft Excel spreadsheet, means and standard deviations calculated. Graphical presentation of results was developed in Excel. In vitro fermentation parameters analysis of variance was conducted using SAS General Linear Models^54^ with mean separation by Fisher’s protected least significant difference test at a probability level of 0.05. Pairwise comparisons of *in-vitro* fermentation parameters at initial conditions vs. 24 h incubation were by one-sided *t*-test in SAS.

The raw 16S-rRNA amplicon sequencing data was processed through the QIIME2 (2020.6) pipeline^55^. Briefly, paired end sequence data was de-multiplexed and amplicon sequence variants (ASV) were assigned using the DADA2 plugin^56^. A phylogenetic tree was constructed using FastTree 2^57^. Taxonomy was assigned based on a pre-trained Naive Bayes classifier trained on the Greengenes database (v13.8) for the 16S rRNA gene spanning the V1-V2 region^58^. The between sample diversity (weighted and unweighted UniFrac distances) were computed using the ‘qiime diversity’ plugin.

A nonparametric permutational multivariate ANOVA (PERMANOVA) test^59^ implemented in the vegan package for R was used for beta diversity matrices. Pairwise Wilcoxon Rank Sum Test was used to determine differences in bacterial genera between treatment groups. The P values were adjusted using the Bonferroni correction method. A P value of 0.05 was used to define significance.

## Supplementary Information

Supplementary Information.

## Data Availability

All data associated with this work are available in the supplementary materials.

## Abbreviations

CC: Corn cobs
CS: Corn stalks
C + P: Ground corn and protein mix
FFV: Fresh fruit and vegetables
IUUB: Indigestible, unpalatable, or undesirable biomass
MS: Mushroom stumps
VFAs: Volatile fatty acids
SMC: Spent mushroom compost
TMR: Total mixed ration
WBG: Wet brewers’ grains
