# Supplementary material for: Proof of concept for developing novel feeds for cattle from wasted food and crop biomass to enhance agri-food system efficiency
Source: Sci Rep. 2022 Aug 10;12:13630. doi: 10.1038/s41598-022-17812-w (PMC9365796; doi:10.1038/s41598-022-17812-w)
Supplement: Supplementary file 1 — Supplementary Information. [file 41598_2022_17812_MOESM1_ESM.docx]

Proof of Concept for Developing Novel Feeds for Cattle from Wasted Food and Crop Biomass to Enhance Agri-Food System Efficiency

Zhengxia Dou, John D. Toth, Dipti W. Pitta, Joseph S. Bender, Meagan L. Hennessy, Bonnie Vecchiarelli, Nagaraju Indugu, Ting Chen, Yunyun Li, Rachel Sherman, Jonathan Deutsch, Bo Hu, Gerald C. Shurson, Brianna Parsons, Linda D. Baker

**Supplementary Table 1**. Ensiling studies in terms of substrates, relevant proportions, and experiment duration.

| Trial-Treatment ^1^ | Weight %  (as-is basis) | Dry Matter (%) | Duration (days) |
| --- | --- | --- | --- |
|  |  |  |  |
| **Trial 1, three treatments** |  |  |  |
| FFV alone | 100.0 | 12.3 | 42 |
| FFV+CC | 81.7+18.3 | 25.3 | 42 |
| FFV+SMC | 62.6+37.4 | 25.1 | 42 |
|  |  |  |  |
| **Trial 2, one treatment** |  | 24.5 | 42 |
| FFV | 46.7 |  |  |
| MS | 9.2 |  |  |
| CS | 8.2 |  |  |
| SMC | 10.0 |  |  |
| WBG | 25.9 |  |  |
|  |  |  |  |
| **Trial 3, two treatments** |  |  |  |
| FFV+ CS | 82.9+17.1 | 24.4 | 28 |
| FFV+ CS +WBG | 57.8+13.8+28.4 | 24.7 | 28 |

^1^ Abbreviations: FFV, fresh fruit and vegetables; CC, corn cobs; SMC, spent mushroom compost; MS, mushroom stumps; CS, corn stalks; WBG, wet brewers’ grains.

**Supplementary Table 2**. Physical, chemical, and nutritional analyses of raw substrates used in ensiling experiments. ^1^

| Analysis | FFV | CC | CS | SMC | MS | WBG |
| --- | --- | --- | --- | --- | --- | --- |
| Dry matter (%) | 12.8 | 82.6 | 88.0 | 47.0 | 9.9 | 20.6 |
| pH | 4.2 | 6.37 | 7.18 | 7.02 | 6.52 | 5.10 |
| Adjusted protein (% DM) | 10.1 | 7.1 | 4.9 | 15.3 | 24.6 | 39.2 |
| Adjusted protein (% CP) | − | 6.4 | 3.5 | 13.3 | 21.9 | 36.8 |
| Soluble protein (% DM) | 6.6 | 89.8 | 71.7 | 86.9 | 89.2 | 93.8 |
| Soluble protein (% CP) | 65.0 | 2.3 | 1.3 | 4.0 | 10.2 | 4.1 |
| ADF protein (% DM) | − | 31.6 | 26.4 | 26.2 | 41.7 | 10.5 |
| ADF protein (% CP) | − | 1.21 | 1.89 | 3.34 | 4.42 | 4.04 |
| NDF protein (% DM) | − | 17.0 | 38.3 | 21.8 | 18.0 | 10.3 |
| NDF protein (% CP) | − | 1.66 | 1.94 | 14.38 | 9.31 | 4.35 |
| ADF (% DM) | 5.7 | 23.3 | 39.4 | 93.9 | 37.9 | 11.1 |
| ADF (% NDF) | − | 43.6 | 55.5 | 31.3 | 15.7 | 23.4 |
| aNDF (% DM) | − | 60.7 | 67.3 | 82.2 | 51.1 | 55.3 |
| aNDF, ash-free (% DM) | − | 71.8 | 82.5 | 41.4 | 30.7 | 42.4 |
| Lignin (% DM) | 1.48 | − | − | 32.6 | − | − |
| Lignin (% NDF) | 14.17 | 5.39 | 8.68 | 25.79 | 2.45 | 8.06 |
| Water-sol. carb. (% DM) | 25.37 | 7.51 | 10.52 | 1.8 | 7.97 | 19.0 |
| Ethanol-sol. carb. (% DM) | − | 2.9 | − | 1.8 | 8.8 | − |
| Ethanol-sol. carb. (% NFC) | − | 1.4 | 1.7 | 0.8 | 3.5 | 1.3 |
| Starch (% DM) | − | 8.6 | 24.6 | 2.6 | 8.1 | 23.7 |
| Starch (% NFC) | − | 2.4 | 3.5 | 0.1 | 2.5 | 0.5 |
| Crude fat (% DM) | − | 14.9 | 50.8 | 0.2 | 5.9 | 9.7 |
| Ash (% DM) | 5.89 | 4.83 | 6.84 | 30.15 | 9.03 | 5.44 |
| Calcium (% DM) | 0.19 | 0.31 | 0.30 | 5.62 | 0.45 | 0.48 |
| Phosphorus (% DM) | 0.26 | 0.14 | 0.07 | 0.68 | 0.74 | 0.60 |
| Magnesium (% DM) | 0.14 | 0.14 | 0.12 | 0.57 | 0.09 | 0.31 |
| Potassium (% DM) | 2.48 | 0.72 | 1.00 | 2.49 | 3.39 | 0.13 |
| Sulfur (% DM) | − | 0.10 | 0.07 | 1.77 | 0.30 | 0.45 |
| Sodium (% DM) | 0.08 | 0.03 | 0.01 | 0.58 | 0.18 | 0.10 |
| Chloride (% DM) | − | 0.18 | 0.07 | 0.65 | 0.53 | 0.05 |
| Iron (% DM) | 88 | 1277 | 1241 | 2485 | 326 | 315 |
| Manganese (% DM) | 37 | 52 | 100 | 379 | 24 | 80 |
| Zinc (% DM) | 30 | 45 | 24 | 204 | 48 | 103 |
| Copper (% DM) | 14 | 21 | 8 | 165 | 30 | 31 |
| Total digestible nutrients (% DM) | − | 55.8 | 44.0 | 33.7 | 67.8 | 71.9 |
| Net energy lactation (Mcal lb^-1^) | − | 0.57 | 0.44 | 0.32 | 0.70 | 0.74 |
| Net energy maintenance (Mcal lb^-1^) | − | 0.54 | 0.30 | 0.08 | 0.76 | 0.83 |
| Net energy gain (Mcal lb^-1^) | − | 0.28 | 0.06 | -0.15 | 0.48 | 0.54 |
| Metabolizable energy (Mcal lb^-1^) | − | 0.92 | 0.69 | 0.48 | 1.17 | 1.25 |
| NDF digestion rate (Kd, % HR) | − | − | 3.0 | − | − | − |
| Relative feed value | − | − | 51 | − | − | − |
| Non-fiber carbohydrates (% DM) | − | 16.35 | 43.15 | 30.26 | 43.15 | 5.5 |
| Non-structural carbohydrates (% DM) | − | 3.8 | 6.0 | 0.9 | 6.0 | 1.8 |
| DCAD (meq 100 g^-1^) | − | 8.4 | 60.5 | -40.2 | 60.5 | -22.1 |

^1^ Abbreviations: FFV, fresh fruits and vegetables; CC, corn cobs; SMC, spent mushroom compost; CS, corn stalks; MS, mushroom stumps; WBG, wet brewers’ grains.

**Supplementary Table 3**. Nutritional analyses for selected samples from ensiling experiments.^1^

| Nutritional parameters | --------FFV------- | | --------FFV+CC------ | | ---------FFV+SMC-------- | | FFV+MS+CS+SMS+WBG | | FFV+CS | FFV+CS+WBG |
| --- | --- | --- | --- | --- | --- | --- | --- | --- | --- | --- |
|  | D3 | D42 | D3 | D42 | D3 | D42 | D0 | D42 | D28 | D28 |
| Dry matter (%) | 12.9±0.1 | 11.6±1.4 | 25.6±0.5 | 23.6±0.3 | 27.2±1.3 | 22.9±0.8 | 24.5±0.5 | 20.7±0.2 | 23.4±0.9 | 22.0±0.9 |
| pH | 4.19±0.01 | 3.71±0.02 | 4.20±0.02 | 3.88±0.01 | 4.48±0.07 | 4.00±0.03 | 5.64±0.04 | 4.08±0.51 | 3.91±0.01 | 4.42±0.09 |
| Adj. prot. (% DM) | − | 10.5 | − | 7.4 | − | 13.8 | 15.3±0.3 | 18.1±0.5 | 6.9±0.6 | 15.3±1.0 |
| Adj. prot. (% CP) | − | − | − | − | − | − | 90.0±0.2 | 16.5±0.2 | 90.6±0.5 | 93.7±0.5 |
| Sol. Prot. (% DM) | 6.6±0.3 | 6.6±0.5 | 3.2±0.1 | 3.8±0.8 | 4.5±0.3 | 4.2±0.2 | 3.5±0.2 | 4.9±0.6 | 3.1±0.2 | 5.2±0.1 |
| Sol. prot. (% CP) | 65.0±1.5 | 63.1±1.0 | 48.4±0.7 | 50.4±0.7 | 32.1±1.9 | 30.2±0.7 | 20.6±0.6 | 27.2±2.0 | 41.2±2.1 | 32.6±0.2 |
| NH_3_ (% DM) | − | − | − | − | − | − | 0.11±0.05 | 1.57±0.92 | 0.79±0.06 | 1.57±0.09 |
| NH_3_ (% CP) | − | − | − | − | − | − | 0.63±0.23 | 8.53±4.74 | 10.50±0.30 | 9.87±0.29 |
| NH_3_ (% SP) | − | − | − | − | − | − | 3.00±1.13 | 30.67±14.78 | 25.53±1.68 | 30.27±0.99 |
| ADF prot. (% DM) | − | − | − | − | − | − | 2.83±0.12 | 27.2±0.04 | 1.19±0.06 | 1.65±0.07 |
| ADF prot. (% CP) | − | − | − | − | − | − | 16.63±0.31 | 2.70±0.32 | 15.7±0.8 | 10.3±0.8 |
| NDF prot. (% DM) | − | − | − | − | − | − | 3.34±0.42 | 4.06±0.33 | 1.36±0.03 | 1.86±0.09 |
| NDF prot. (% CP) | − | − | − | − | − | − | 19.7±2.9 | 22.5±2.6 | 18.1±1.3 | 11.7±0.8 |
| ADF (% DM) | 5.7±0.4 | 13.6±1.7 | 33.6±0.6 | 36.5±2.5 | 25.1±0.1 | 20.4±12.2 | 36.6±1.8 | 39.2±0.9 | 40.4±1. 9 | 37.7±4.0 |
| ADF (% NDF) | 54. ±3.4 | 85.1±3.4 | 57.9±3.0 | 63.7±2.2 | 82.9±1.7 | 61.9±35.7 | 78.6±1.9 | 81.7±2.4 | 70.0±3.1 | 68.0±3.4 |
| aNDF (% DM) | 10.5±0.3 | 15.9±1.6 | 58.2±2.6 | 57.2±2.0 | 30.2±0.6 | 32.4±1.6 | 46.6±2.5 | 48.0±0.7 | 57.8±2.8 | 55.4±3.4 |
| Lignin (% DM) | 1.48±0.04 | 2.32±0.33 | 4.24±0.34 | 4.82±0.45 | 15.49±0.21 | 18.70±0.58 | 9.78±0.27 | 10.12±1.65 | 6.42±0.47 | 6.38±0.83 |
| Lignin (% NDF) | 14.2±0.86 | 15.6±2.11 | 7.3±0.52 | 8.4±0.67 | 51.2±1.69 | 57.8±1.48 | 21.03±0.56 | 21.09±3.55 | 11.11±0.37 | 11.49±0.84 |
| Water-sol. carb. (% DM) | 25.4±2.1 | − | 8.0±0.0 | − | 6.0±0.8 | − | − | − | − | − |
| Ethanol-sol. carb. (% DM) | − | − | − | − | − | − | 7.27±0.42 | 1.33±0.12 | 1.00±0.75 | 0.53±0.45 |
| Ethanol-sol. carb. (% NFC) | − | − | − | − | − | − | 28.43±2.70 | 5.80±0.36 | 3.80±2.95 | 2.63±1.86 |
| Silage acids (% DM) | − | − | − | − | − | − | 2.77±0.38 | 5.03±2.12 | 10.13±0.56 | 8.40±0.89 |
| Silage acids (% NDF) | − | − | − | − | − | − | 10.57±0.23 | 21.73±8.63 | 38.70±0.56 | 46.70±0.08 |
| Starch (% DM) | − | − | − | − | − | − | 5.37±1.59 | 5.07±3.41 | 6.90±1.91 | 8.37±1.10 |
| Starch (% NFC) | − | − | − | − | − | − | 20.53±3.37 | 21.67±13.98 | 26.27±6.73 | 45.70±3.40 |
| Crude fat (% DM) | − | − | − | − | − | − | 3.43±0.29 | 3.90±0.42 | 1.60±0.31 | 4.16±0.43 |
| Ash (% DM) | 5.89±0.39 | 7.96±0.66 | 4.25±0.47 | 3.84±0.59 | 21.29±0.36 | 21.47±0.51 | 10.57±0.40 | 10.94±0.62 | 8.32±0.51 | 7.98±0.86 |
| Calcium (% DM) | 0.19±0.02 | 0.25±0.03 | 0.19±0.01 | 0.28±0.06 | 3.73±0.12 | 3.66±0.09 | 1.37±0.01 | 1.36±0.05 | 0.29±0.01 | 0.35±0.06 |
| Phosphorus (% DM) | 0.26±0.02 | 0.26±0.02 | 0.15±0.03 | 0.17±0.02 | 0.57±0.02 | 0.57±0.02 | 0.39±0.01 | 0.40±0.00 | 0.16±0.01 | 0.30±0.02 |
| Magnesium (% DM) | 0.14±0.01 | 0.14±0.02 | 0.11±0.01 | 0.14±0.02 | 0.45±0.01 | 0.58±0.23 | 0.26±0.01 | 0.26±0.00 | 0.15±0.00 | 0.18±0.01 |
| Potassium (% DM) | 2.48±0.20 | 2.42±0.33 | 1.34±0.05 | 1.35±0.08 | 2.60±0.04 | 2.67±0.13 | 1.29±0.05 | 1.49±0.02 | 1.59±0.03 | 1.28±0.06 |
| Sulfur (% DM) | − | − | − | − | − | − | 0.56±0.03 | 0.62±0.02 | 0.12±0.01 | 0.22±0.01 |
| Sodium (% DM) | 0.08±0.01 | 0.09±0.02 | 0.05±0.02 | 0.05±0.01 | 0.44±0.01 | 0.45±0.04 | 0.17±0.01 | 0.18±0.01 | 0.04±0.01 | 0.05±0.03 |
| Chloride (% DM) | − | − | − | − | − | − | 0.28±0.02 | 0.32±0.02 | 0.18±0.01 | 0.16±0.02 |
| Iron (% DM) | 88±34 | 101±20 | 406±21 | 460±55 | 1502±94 | 1726±201 | 1304±37 | 1388±66 | 1996±102 | 1465±106 |
| Manganese (% DM) | 37±16 | 19±3 | 35±16 | 24±8 | 247±10 | 251±4 | 142±4 | 135±2 | 126±5 | 105±5 |
| Zinc (% DM) | 30±7 | 28±3 | 34±10 | 32±3 | 148±8 | 151±6 | 72±1 | 78±4 | 28±2 | 60±8 |
| Copper (% DM) | 14±6 | 11±2 | 13±6 | 15±6 | 125±5 | 119±3 | 47±3 | 53±2 | 10±1 | 12±1 |
| TDN(% DM) | − | − | − | − | − | − | 53.4±1.0 | 53±2.2 | 54.9±1.42 | 59.0±3.38 |
| NEL(Mcal lb^-1^) | − | − | − | − | − | − | 0.54±0.01 | 0.54±0.02 | 0.56±0.01 | 0.60±0.04 |
| NEM (Mcal lb^-1^) | − | − | − | − | − | − | 0.49±0.02 | 0.49±0.04 | 0.52±0.03 | 0.60±0.06 |
| Net energy gain (Mcal lb^-1^) | − | − | − | − | − | − | 0.24±0.02 | 0.24±0.04 | 0.26±0.02 | 0.34±0.06 |
| ME(Mcal lb^-1^) | − | − | − | − | − | − | 0.88±0.02 | 0.89±0.05 | 0.91±0.03 | 0.99±0.07 |
| NDF digestion rate (Kd, % HR) | − | − | − | − | − | − | − | − | − | − |
| Relative feed value | − | − | − | − | − | − | − | − | − | − |
| NFC(% DM) | − | − | − | − | − | − | 25.7±3.3 | 23.1±0.70 | 26.1±1.44 | 18.4±3.89 |
| NSC (% DM) | − | − | − | − | − | − | 12.63±1.93 | 6.40±3.18 | 7.90±2.57 | 8.90±1.55 |
| DCAD (meq 100 g^-1^) | − | − | − | − | − | − | 2.33±0..87 | 1.81±1.65 | 29.73±0.75 | 16.93±1.72 |

^1^ Abbreviations: FFV, fresh fruits and vegetables; CC, corn cobs; SMC, spent mushroom compost; CS, corn stalks; MS, mushroom stumps; WBG, wet brewers’ grains.

**Supplementary Table 4**. Description of treatments in the *in-vitro* incubation experiment.^1^

| Treatment | | TMR | NovelFeed-1 | NovelFeed-2 | Ground Corn | | Protein Mix |
| --- | --- | --- | --- | --- | --- | --- | --- |
|  |  | (g) | | | | | |
| 1 | Control | 0.7500 |  |  |  | |  |
| 2 | TMR+NovelFeed-1@5% | 0.7125 | 0.0375 |  |  | |  |
| 3 | TMR+NovelFeed-1@10% | 0.6750 | 0.0750 |  |  | |  |
| 4 | TMR+NovelFeed-1@10% +C+P | 0.5612 | 0.0750 |  | 0. 0603 | | 0.0536 |
|  |  |  |  |  |  | |  |
| 5 | TMR+NovelFeed-2@5% | 0.7125 |  | 0.0375 |  | |  |
| 6 | TMR+NovelFeed-2@10% | 0.6750 |  | 0.0750 |  |  | |
| 7 | TMR+NovelFeed-2@10% +C+P | 0.5994 |  | 0.0750 | 0.0469 | 0.0288 | |

^1^ Abbreviations: TMR, total mixed ration; C+P, ground corn and protein mix.

**Supplementary Table 5**. Pairwise comparison of *in vitro* fermentation parameters at two sampling times (0 and 24 hrs incubation). Values are probability levels using the *t*-test (Pr > |*t*|).

|  | pH | NH_3_-N | Acetic acid | Propionic acid | Butyric acid | Isobutyric acid | Isovaleric acid | Valeric acid |
| --- | --- | --- | --- | --- | --- | --- | --- | --- |
| Diet |  | mg dL^-1^ | % mmol | | | | | |
|  |  |  |  |  |  |  |  |  |
| TMR | <0.0001 | 0.0120 | <0.0001 | <0.0001 | 0.0001 | 0.0008 | 0.0001 | <0.0001 |
| TMR+NovelFeed1@5% | <0.0001 | 0.6544 | 0.0109 | 0.0031 | 0.0338 | 0.3804 | 0.2790 | 0.0044 |
| TMR+ NovelFeed1@10% | <0.0001 | 0.0238 | 0.0023 | 0.0014 | 0.0109 | 0.0662 | 0.1638 | <0.0001 |
| TMR+ NovelFeed2@5% | <0.0001 | 0.0079 | 0.0002 | <0.0001 | 0.0007 | 0.7552 | 0.0209 | 0.0004 |
| TMR+ NovelFeed2@10% | <0.0001 | 0.0774 | 0.0108 | 0.0042 | 0.0432 | 0.0233 | 0.1109 | 0.0029 |
| TMR+ NovelFeed1@10%+C+P | <0.0001 | 0.1570 | 0.0010 | 0.0001 | 0.0141 | 0.4461 | 0.2183 | 0.0012 |
| TMR+ NovelFeed2@10%+C+P | <0.0001 | 0.0926 | 0.0007 | <0.0001 | 0.0063 | 0.1075 | 0.4080 | 0.0024 |

**Supplementary Table 6**. Comparison of individual bacterial genera at day 24 (Top 5 genera selected from the top phylum)

|  | TMR+  5%NF1 | TMR+  10%NF1 | TMR+  10%NF1+C+P | TMR+  5%NF2 | TMR+  10%NF2 | TMR+  10%NF2+C+P | TMR |
| --- | --- | --- | --- | --- | --- | --- | --- |
| Bacteroidetes Prevotella | 28.07 | 25.93 | 26.18 | 27.07 | 25.35 | 24.58 | 30.12 |
| Bacteroidetes unclassified S24-7 | 2.57 | 2.73 | 2.62 | 2.25 | 2.37 | 3.32 | 2.36 |
| Bacteroidetes unclassified Bacteroidales | 2.60 | 2.79 | 1.84 | 2.60 | 2.97 | 2.43 | 1.86 |
| Bacteroidetes unclassified Paraprevotellaceae | 1.67 | 1.68 | 1.51 | 1.90 | 1.98 | 1.78 | 1.80 |
| Bacteroidetes unclassified Prevotellaceae | 1.07 | 1.20 | 0.93 | 1.16 | 1.27 | 0.98 | 0.85 |
| Fibrobacteres Fibrobacter | 1.03 | 1.29 | 0.46 | 1.14 | 1.50 | 0.74 | 0.69 |
| Firmicutes Butyrivibrio | 5.52 | 5.78 | 7.28 | 5.79 | 6.06 | 7.12 | 5.61 |
| Firmicutes unclassified Clostridiales | 5.66 | 5.57 | 6.51 | 5.31 | 5.46 | 6.02 | 5.13 |
| Firmicutes unclassified Clostridiales | 5.74 | 4.88 | 5.54 | 4.91 | 5.02 | 5.51 | 5.49 |
| Firmicutes Ruminococcus | 5.09 | 5.36 | 4.33 | 5.04 | 5.45 | 4.88 | 4.40 |
| Proteobacteria unclassified Succinivibrionaceae | 2.50 | 2.77 | 1.57 | 3.20 | 2.48 | 1.47 | 2.58 |
| Proteobacteria Ruminobacter | 0.61 | 0.70 | 0.43 | 0.93 | 0.67 | 0.48 | 0.68 |
| Proteobacteria Succinivibrio | 0.29 | 0.36 | 0.24 | 0.42 | 0.43 | 0.23 | 0.40 |
| Spirochaetes Treponema | 3.45 | 4.57 | 2.44 | 4.17 | 4.71 | 2.89 | 3.22 |
| Tenericutes Anaeroplasma | 0.66 | 0.83 | 0.88 | 0.66 | 0.77 | 0.63 | 0.65 |
| Tenericutes unclassified Mollicutes | 0.28 | 0.32 | 0.23 | 0.33 | 0.29 | 0.15 | 0.28 |
